# Supplementary material for: “Running on goodwill and fairydust” – Midwives' experiences of facilitating and delivering local breastfeeding support via Facebook groups: A qualitative descriptive study
Source: J Hum Nutr Diet. 2024 Oct 23;38(1):e13367. doi: 10.1111/jhn.13367 (PMC11589396; doi:10.1111/jhn.13367)
Supplement: Supplementary file 1 — Supporting information. [file JHN-38-0-s001.docx]

Interview Guide: A semi-structured interview schedule including motivations for BSF group facilitation, format and perceived value

**Background**

- What is your professional role and remit?
- Are you involved in using a Facebook group to provide local breastfeeding support?

**Local BSF group role**

- How would describe your current role within/in relation to the BSF group?
  - How long do you spend each week on this role? Is this time paid?
  - Have you had any special training for this role in breastfeeding or online support? Do you think this is needed?
- How did you become involved in BSF group provision?
  - Did you have any concerns about doing so? How did you resolve these?

**Local BSF group format and function**

- If applicable, can you describe the process of setting up the group?
  - Were there any issues? How were these resolved?
  - Do you have any written guidelines?
- Can you tell me about the format of your group and who contributes to its function?
  - Does the group collaborate with any non-health professionals who offer BSF support?
  - Does it have a connected face to face support group/s? Any other local links?
- How would you describe the purpose of local Facebook group support for breastfeeding?
  - What do you think are the benefits for mothers? Do you have any concerns?

**Support and experiences**

- How are you and the group supported by the local health board/trust?
- Can you describe any personal or professional challenges you have faced in your role?
  - How did you manage these?
- Has being involved in the group had any positive impacts on you?
  - Can you tell me about these?
- What attitudes have you encountered about the provision of BSF groups?
  - Within maternity services? From midwives?
  - Have you seen any change since the COVID-19 outbreak?
- What are your thoughts on all maternity services providing BSF groups?
  - What are the barriers to more midwives getting involved?
- Is there anything else you’d like to comment on/add?
